# Huntington's Disease Research Over Six Decades: Global Insights, Gaps, and Future Directions

**DOI:** 10.7759/cureus.97567

**Published:** 2025-11-23

**Authors:** Hasini Masna, Meghana Konda, Latha Ganti

**Affiliations:** 1 Neurology, Flower Mound High School, Dallas, USA; 2 Public Health, Brown University, Providence, USA; 3 Emergency Medicine and Neurology, University of Central Florida, Orlando, USA; 4 Medical Science, The Warren Alpert Medical School of Brown University, Providence, USA

**Keywords:** bibliometric analysis, genetic testing, global research trends, huntington’s disease, neurodegenerative disorders

## Abstract

Huntington’s disease (HD) is a hereditary neurodegenerative disorder caused by expanded cytosine-adenine-guanine (CAG) repeats in the huntingtin (HTT) gene. It leads to progressive decline in motor function, cognition, and behavior, often following a prolonged pre-symptomatic phase. Although research on HD has progressed, significant gaps remain in understanding its full impact, particularly in areas such as mental health, global collaboration, and early intervention.

A bibliometric analysis was conducted using the Web of Science Core Collection to evaluate global research trends in HD and its testing from 1966 to 2025. A total of 1,515 publications were analyzed for authorship patterns, contributing countries, journal sources, and frequently occurring keywords. VOSviewer software v1.6.15 (Centre for Science and Technology Studies, Leiden University, The Netherlands) was used to visualize author networks and keyword co-occurrence.

Publication activity peaked in 2014 and 2018, with 101 and 96 articles published, respectively. The United States emerged as the leading contributor to HD research, followed by European countries with fewer publications. Keyword analysis revealed strong associations between HD and other neurodegenerative disorders, such as Alzheimer’s and Parkinson’s disease, as well as recurring terms related to genetic testing, brain anatomy, and animal models. Limited author collaboration was observed, with only a few dense research clusters present.

This analysis highlights the growing body of research on HD, particularly in genetic mechanisms and therapeutic modeling. However, the concentration of research within a few countries and author groups suggests limited global collaboration. Emerging gaps include underrepresentation of mental health impacts, disparities in geographic research output, and narrow journal dissemination. Strengthening international cooperation and diversifying research focus could accelerate progress in diagnosis, treatment, and overall patient care.

## Introduction and background

Huntington’s disease (HD), a progressive neurodegenerative disorder of the central nervous system, has a prevalence of 4.88 per 100,000 individuals and is inherited in an autosomal dominant pattern. Patients commonly present with chorea, incoordination, cognitive decline, dystonia, and disruptive behavior disorder [1,2]. HD is caused by expansion of cytosine-adenine-guanine (CAG) trinucleotide repeats in the huntingtin (HTT) gene on chromosome 4, producing an abnormal protein with an extended polyglutamine tract [3]. Individuals with >39 CAG repeats are highly likely to develop HD, whereas 36-39 repeats are associated with reduced penetrance [4,5]. Intermediate alleles can expand intergenerationally, particularly with paternal transmission, resulting in a disease-causing repeat length in offspring [6].

Although individuals may appear healthy preclinically, cognitive, behavioral, and motor changes can emerge during a prediagnostic phase [1]. Because of this, an interdisciplinary approach and early intervention are critical. From 1985 to 2010, the average incidence was 0.38 per 100,000 person-years [7]. On average, there were 0.38 new cases of HD for every 100,000 people per year from studies conducted between 1985 and 2010 [8]. In European populations, HD is relatively common, affecting about 10 to 13 people per 100,000. It is much rarer in East Asia, with only one to seven cases per million. In South Africa, Black individuals are less likely to have the disease compared to White or mixed-ancestry groups. Current research trends are moving from discovery to personalization, such as early detection via biomarkers and imaging, precision therapeutics including stem cells, and developmental timing to identify HD before symptom onset [9]. Nonetheless, research gaps persist in mental health and global disparities as they relate to HD.

Despite this growth, evidence remains limited regarding health-care utilization, service needs, and health-related quality of life [10]. Therapeutic areas gaining attention include antisense oligonucleotide (ASO), RNA interference (RNAi), stem-cell therapies, antibody therapies, and expanded preclinical development pipelines [11,12].

Given persistent gaps, ongoing work must address the causes, manifestations, and testing of HD and, importantly, patient-centered outcomes. A bibliometric review is important for this field as it allows one to characterize publishing trends across countries, journals, and keywords to clarify where research is concentrated and where it is absent.

## Review

Methods

Data Source and Search Strategy

This study utilized data from the Web of Science (WOS) Core Collection, a comprehensive and widely used database that indexes a vast array of scholarly articles spanning from the late 20th century to the present. Due to its inclusion of high-quality publications and its robust citation-tracking capabilities, WOS is considered a preferred source for bibliometric research [11].

We queried the WOS Core Collection for publications on HD and testing from January 1, 1966, through June 30, 2025 (inclusive). Searches were run in the Topic field (Title, Abstract, Author Keywords, and Keywords Plus). The primary Boolean query was: ("Huntingtons" AND "testing"). No language, document type, or country filters were applied at retrieval to maximize sensitivity. Exports used “Full record and cited references” in batches of 500.

The inclusion criteria for this study were peer-reviewed publications addressing HD and any form of clinical or genetic testing (diagnostic, predictive, confirmatory, and counseling-related testing) as ascertainable from the title/abstract/keywords. The exclusion criteria were publications outside 1966-2025, non-article records if clearly editorial, news, or a meeting abstract without analyzable metadata, and studies centered on unrelated genetic disorders without a substantive HD testing component. Duplicates were removed using WOS unique identifiers and DOI matching.

Data Analysis

A bibliometric approach quantified publication patterns and thematic developments [13]. Text-delimited WOS files were imported into VOSviewer v1.6.15 (Centre for Science and Technology Studies, Leiden University, The Netherlands) for processing and visualization.

VOSviewer was used to identify leading contributors, including the most frequently cited authors, journals, and recurring keywords. These elements were visualized using bibliometric maps, which illustrate bibliographic coupling by displaying how often articles, authors, or journals are cited together within the same body of literature [14].

The visualizations showed the strength of connections with terms such as author names, countries, journal titles, and keywords. These terms are interconnected by lines, with the length and thickness of each line representing the degree of relatedness or co-occurrence between them.

Results

**Figure 1:**
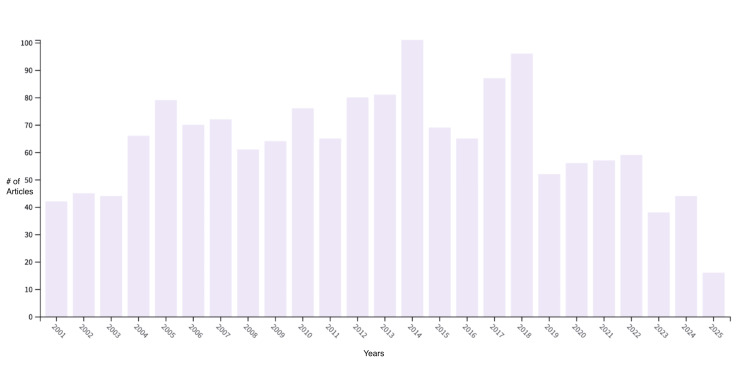
Bar graph of how many papers were published on Huntington’s disease and testing over the past 25 years

A total of 1,515 publications were identified during this period (Figure 1). The first notable increase in research activity occurred in 2005, with 79 papers published. The peak year for publications was 2014, which saw the highest output with 101 articles. Following this, the number of publications declined, reaching a low point in 2017 and 2018 with 87 and 96 publications, respectively. The years with the fewest publications were 2001 (42 articles, 2.8%), 2003 (44 articles, 2.9%), and 2023 (38 articles, 2.5%). Because 2025 indexing is incomplete, 2025 items were not included in annual trend tallies. These temporal shifts suggest surges aligned with therapeutic and mechanistic advances, followed by a broad redistribution of research attention.

**Figure 2:**
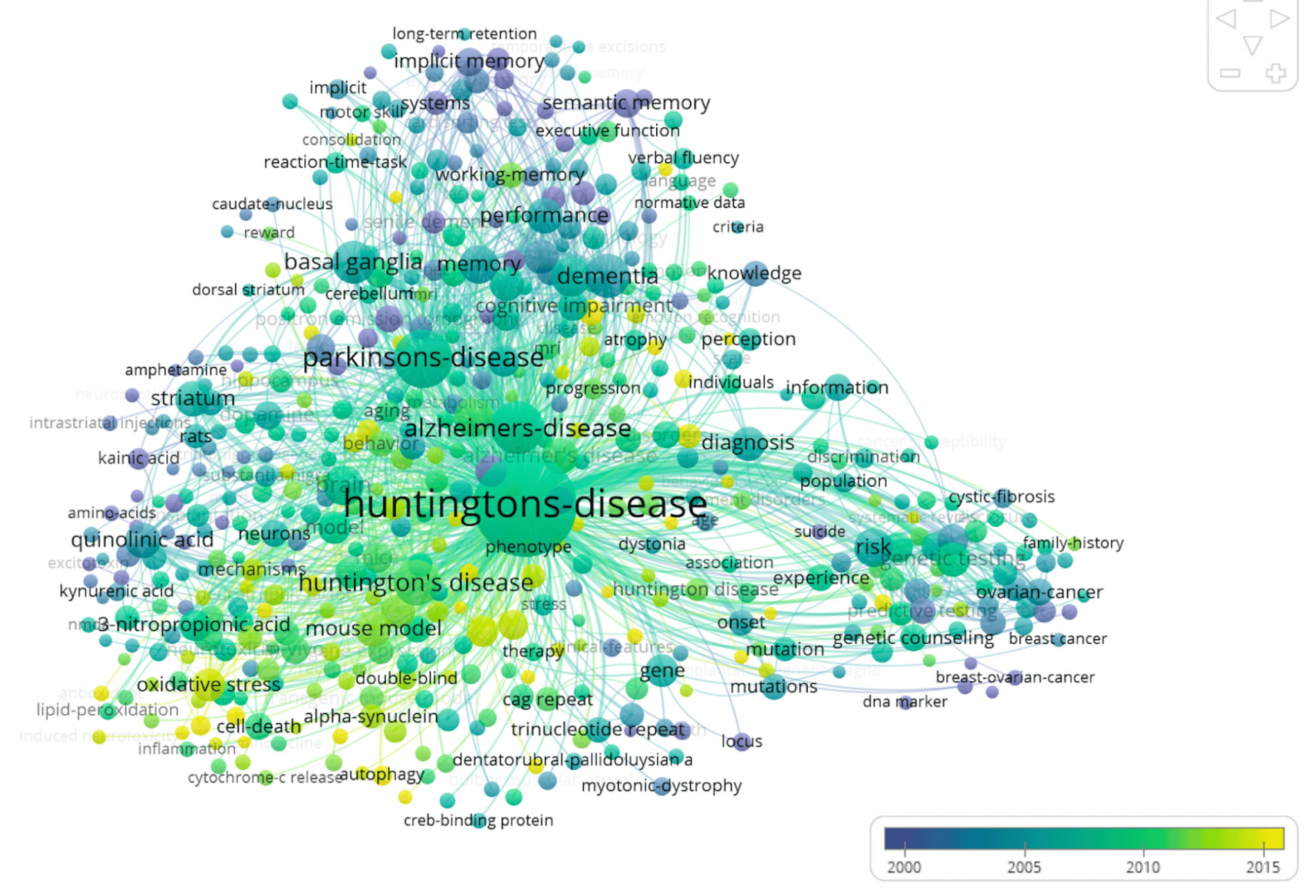
Visual representation of the keywords most commonly associated with studies related to Huntington’s disease and testing

Keyword co-occurrence (1991-2025) revealed dense clustering around neurodegeneration and experimental systems. Frequently co-occurring terms included “Parkinson’s disease” (309), “Alzheimer’s disease” (242), “dementia” (147), “basal ganglia/memory” (138), “striatum” (80), “mouse model” (83), and “3-nitropropionic acid” (58). Less frequent terms were “genetic counseling” (39), “breast-ovarian-cancer” (10), and “oxidative stress” (62), which suggest counseling/ethical and oxidative biology themes (Figure 2).

**Figure 3:**
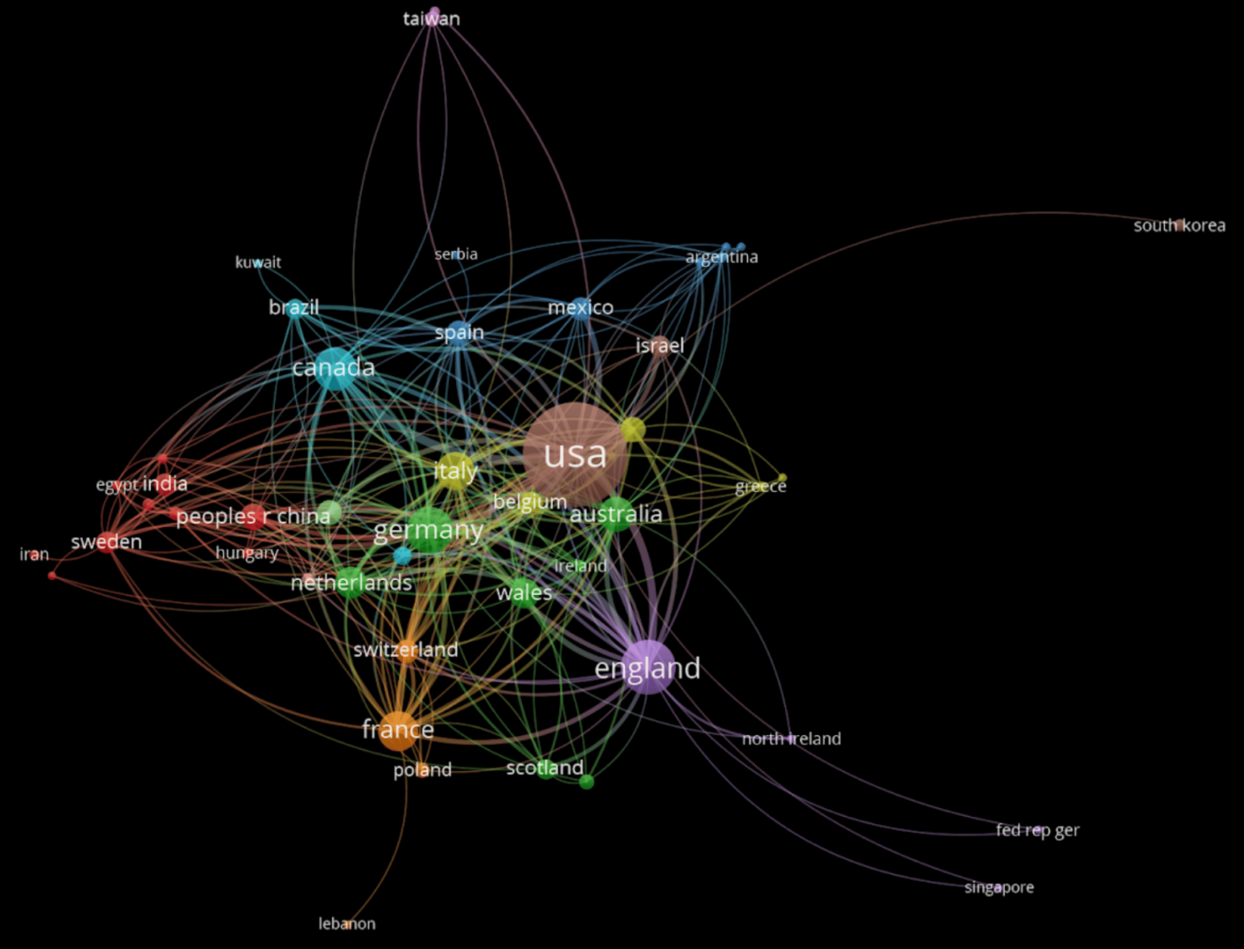
Visual illustration of co-authorship between countries

Figure 3 displays the geographic spread of published materials across the world on HD and testing from 1966 to the present day. In this bibliometric map, the size of each circle indicates the number of articles published by the country. The lines connecting the circles represent co-authorship relationships and are grouped into clusters, each shown in a different color. The thickness of the lines reflects the strength of international collaboration and the degree of connectivity between countries. In this case, the United States demonstrated the strongest international collaboration, with a link strength of 769. Other countries like England (approximately 185), Germany (approximately 150), and Canada (approximately 107) also demonstrate extensive collaboration. The strongest bilateral connection was the United States-England (link strength 29), indicating frequent co-authorship. Despite these ties, cross-regional integration remained uneven, with fewer links among underrepresented regions.

**Figure 4:**
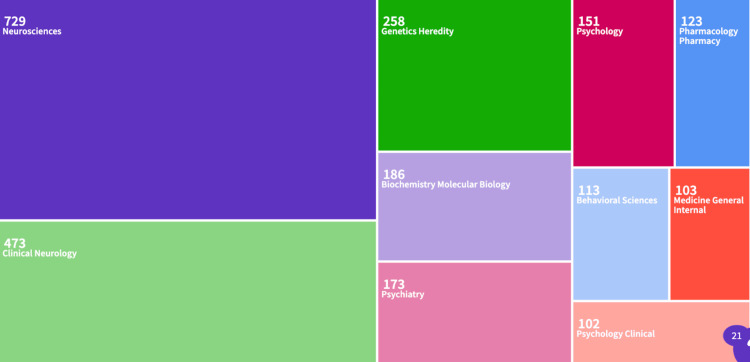
Visual representation of the categories under which Huntington’s disease research falls

Based on the dataset, the majority of papers were published in the field of neurosciences (729), followed by clinical neurology (473). Other areas included genetic heredity (258), biochemistry molecular biology (186), and psychiatry (173). Psychology (151), pharmacology/pharmacy (123), behavioral sciences (113), and general/internal medicine (103) were less represented, signaling limited interdisciplinary breadth (Figure 4).

**Figure 5:**
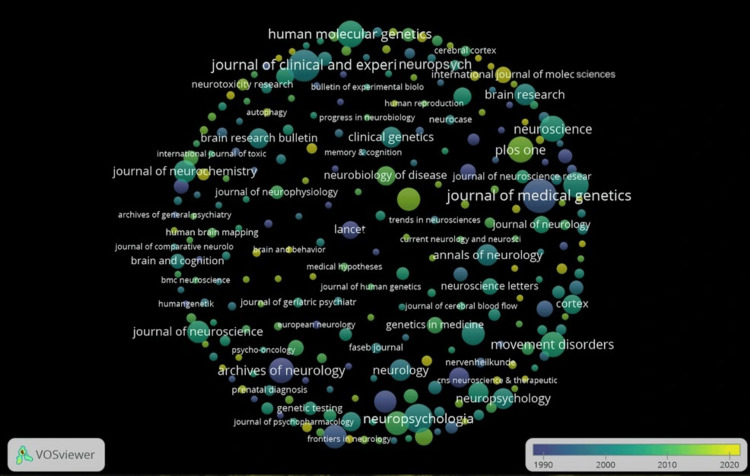
Visual depiction of journals that publish papers on Huntington’s disease and testing

The visualization shows the distribution of publications across journals in the field, with each circle representing a different journal. The size of each circle corresponds to the number of publications related to the topic. Larger circles indicate journals that have published a higher amount of research. For instance, journals such as Journal of Medical Genetics, Journal of Clinical and Experimental Neuropsychology, and Archives of Neurology appear prominently due to their larger size, which signifies their central role in disseminating research on the subject (Figure 5).

**Figure 6:**
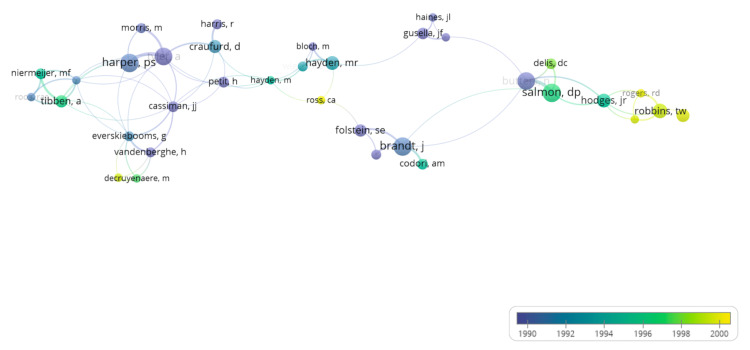
Displays the collaborative connections and efforts between authors

Figure 6 is a visual representation of when and how much authors collaborate and connect with each other. Authors represented by larger circles have published more papers, whereas those in smaller circles have contributed less. The lines represent interconnectedness between authors. The thicker the line, the more collaboration, associations, and citations between them. The clusters show how closely related the authors are to each other. This topology indicates limited inter-cluster connectivity, consistent with siloed research streams. Siloed research streams are not desirable because they lead to inefficiency, poor decision-making, and a lack of innovation. Operating in isolation creates fragmented data, hinders collaboration, and can result in duplicated efforts and inconsistent data quality.

Discussion

HD is a progressive neurodegenerative disorder with significant implications for affected individuals and their families. Advancing treatment strategies is critical not only for symptom management and slowing disease progression but also for developing a potential cure and expanding our understanding of other neurodegenerative conditions [14]. This bibliometric analysis aims to highlight trends in global HD research by examining publication output, contributing countries, prominent journals and authors, and frequently occurring keywords. The findings underscore the need for continued and diversified research efforts in this area.

An analysis of publication trends revealed peak research activity in 2014 and 2018. The peak in 2014 may be attributed to an increase in clinical trials focusing on pharmacological interventions and outcome measures for HD [15]. Similarly, 2018 marked a significant increase in publications, likely driven by advances in understanding the molecular mechanisms behind HTT-related neurodegeneration [16]. However, a decline in publication volume is observed after 2018, which may reflect a global redirection of research resources during the COVID-19 pandemic [17]. This pattern highlights the vulnerability of rare-disease research to broader shifts in public-health priorities.

Keyword analysis revealed consistent associations between HD and other neurodegenerative diseases such as Parkinson’s and Alzheimer’s, likely due to their shared characteristics, including progressive neuronal loss, genetic risk factors, and protein accumulation in the brain [18,19]. Additional keywords such as “basal ganglia,” “striatum,” and “mouse model” point to a strong emphasis on the motor and anatomical disruptions caused by HD and the experimental systems used to model the disease [2,20]. The frequent appearance of “genetic counseling” emphasizes the role of personalized medicine and ethical considerations in HD care, while the presence of unrelated terms like “breast-ovarian-cancer” suggests shared territory in genetic-testing methodologies rather than disease similarity [21].

Geographic trends indicate that the United States leads HD research, likely influenced by its higher prevalence of cases (approximately 3,707) and increased funding capacity. In contrast, countries such as Northern Ireland, with significantly fewer reported cases (around 180), contribute less research output [2]. This imbalance reflects the direct relationship between disease burden and research investment. A more globally collaborative approach could accelerate progress in diagnostics and therapy. Additionally, most research falls under the neuroscience category, with limited emphasis on the psychological and mental-health impacts of HD. Expanding research into the mental and emotional consequences of the disease could enhance holistic care for patients and their families.

Leading journals in the field include Archives of Neurology and the Journal of Medical Genetics, reflecting the dual focus on neurological degeneration and genetic pathology. Genetic research on HD is largely centered on the CAG trinucleotide repeat in the HTT gene. Individuals with more than 40 repeats are almost certain to develop the disease, whereas those with 27-35 repeats are typically unaffected but may still be at risk for generational transmission [21]. In terms of author collaboration, while a few well-connected groups, such as those associated with Harper PS, Hayden MR, and Salmon DP stand out, overall collaboration remains limited. The sparsity of inter-author connections suggests that HD research remains concentrated within niche groups, with insufficient interdisciplinary or international engagement.

This review reveals interrelated gaps that constrain progress. International collaboration remains limited, especially across low- and middle-income regions, undermining external validity and slowing equitable translation of advances. Additionally, interdisciplinary engagement is sparse, with limited links to psychiatry, psychology, social science, health services, and ethics, which restricts the design of comprehensive testing pathways and impedes direct implementation. Psychological and mental-health dimensions, including depression, anxiety, caregiver strain, and counseling outcomes, are underexamined and insufficiently embedded within testing-focused research agendas. For example, in a cross-sectional study of persons with HD, 33% reported symptoms of depression, yet measurement of these psychological symptoms seldom appears in clinical trials focused on motor or cognitive endpoints [22]. Another example is highlighted by a prospective longitudinal study of HD patient/caregiver pairs: caregiver burden was significantly associated with the patient’s neuropsychiatric symptoms rather than the motor score; yet many HD research programs do not routinely include caregiver strain or interventions for caregiver mental health within their testing protocols [23]. Addressing these deficits through broader global partnerships, cross-disciplinary teams, and routine integration of mental-health measures would substantially strengthen the relevance, scalability, and patient-centered impact of future work.

Limitations

This analysis is subject to several limitations. It relies exclusively on data retrieved from the WoS Collection, which may exclude relevant publications indexed in other major databases such as PubMed, Scopus, or Embase. Additionally, the inclusion criteria were based solely on selected keywords, potentially omitting studies that addressed HD and testing using alternate terminology. The figures and trends discussed are constrained by the available data, and incomplete indexing or missing records may limit the comprehensiveness of the results. Additionally, underrepresentation of authors from smaller institutions or less resourced regions may obscure valuable contributions to the field.

## Conclusions

This bibliometric analysis highlights several actionable levers for the next phase of HD testing. Research priorities should include sustained programs on psychological and mental-health outcomes, counseling efficacy, and patient-reported measures alongside biological testing. Targeting these priorities can shift the field from concentrated and independent activity toward globally collaborative, patient-centered programs that accelerate equitable advances in diagnosis, counseling, and therapeutic readiness for HD.

## References

[1] Walker FO (2007). Huntington’s disease. Lancet.

[2] Medina A, Mahjoub Y, Shaver L, Pringsheim T (2022). Prevalence and incidence of Huntington's disease: an updated systematic review and meta-analysis. Mov Disord.

[3] Andhale R, Shrivastava D (2022). Huntington's disease: a clinical review. Cureus.

[4] Jimenez-Sanchez M, Licitra F, Underwood BR, Rubinsztein DC (2017). Huntington's disease: mechanisms of pathogenesis and therapeutic strategies. Cold Spring Harb Perspect Med.

[5] Ajitkumar A, De Jesus O (2025). Huntington Disease. https://www.ncbi.nlm.nih.gov/books/NBK559166/.

[6] Caron NS, Wright GE, Hayden MR (2025). Huntington disease. https://www.ncbi.nlm.nih.gov/books/NBK1305/.

[7] Ferguson MW, Kennedy CJ, Palpagama TH, Waldvogel HJ, Faull RL, Kwakowsky A (2022). Current and possible future therapeutic options for Huntington's disease. J Cent Nerv Syst Dis.

[8] Quemener AM, Bachelot L, Forestier A, Donnou-Fournet E, Gilot D, Galibert MD (2020). The powerful world of antisense oligonucleotides: from bench to bedside. Wiley Interdiscip Rev RNA.

[9] Ganti L, Persaud NA, Stead TS (2025). Bibliometric analysis methods for the medical literature. Acad Med Surg.

[10] van Eck NJ, Waltman L (2010). Software survey: VOSviewer, a computer program for bibliometric mapping. Scientometrics.

[11] Haskins BA, Harrison MB (2000). Huntington's disease. Curr Treat Options Neurol.

[12] Carlozzi NE, Miciura A, Migliore N, Dayalu P (2014). Understanding the outcomes measures used in Huntington disease pharmacological trials: a systematic review. J Huntingtons Dis.

[13] Caterino M, Squillaro T, Montesarchio D, Giordano A, Giancola C, Melone MA (2018). Huntingtin protein: a new option for fixing the Huntington's disease countdown clock. Neuropharmacology.

[14] Riccaboni M, Verginer L (2022). The impact of the COVID-19 pandemic on scientific research in the life sciences. PLoS One.

[15] (2024). Alzheimer’s, Parkinson’s, and Huntington’s diseases share a common crucial feature: finding suggests that treatment for one disease could work for the other two. https://www.sciencedaily.com/releases/2017/05/170523144123.htm.

[16] Flavin WP, Bousset L, Green ZC (2017). Endocytic vesicle rupture is a conserved mechanism of cellular invasion by amyloid proteins. Acta Neuropathologica.

[17] Matz OC, Spocter M (2022). The effect of Huntington's disease on the basal nuclei: a review. Cureus.

[18] Kaye J, Reisine T, Finkbeiner S (2021). Huntington's disease mouse models: unraveling the pathology caused by CAG repeat expansion. Fac Rev.

[19] Nance MA (2017). Genetic counseling and testing for Huntington's disease: a historical review. Am J Med Genet B Neuropsychiatr Genet.

[20] Manahan ER, Kuerer HM, Sebastian M (2019). Consensus guidelines on genetic' testing for hereditary breast cancer from the American Society of Breast Surgeons. Ann Surg Oncol.

[21] Myers RH (2004). Huntington's disease genetics. NeuroRx.

[22] Pérez-Pérez J, García-López S, Valle TF (2025). Huntington disease health related quality of life, function and well being: the patient's perspective. Neurol Ther.

[23] Carney S, Pender N, Rogers E (2025). Huntington's disease caregivers: a qualitative exploration of caregivers experience. J Health Psychol.

